# Early clinical signs in neonates with hypoxic ischemic encephalopathy predict an abnormal amplitude-integrated electroencephalogram at age 6 hours

**DOI:** 10.1186/1471-2431-13-52

**Published:** 2013-04-10

**Authors:** Alan R Horn, George H Swingler, Landon Myer, Lucy L Linley, Moegammad S Raban, Yaseen Joolay, Michael C Harrison, Manigandan Chandrasekaran, Natasha R Rhoda, Nicola J Robertson

**Affiliations:** 1 School of Child and Adolescent Health, University of Cape Town, Red Cross War Memorial Children’s Hospital, Klipfontein Rd, Cape Town, South Africa; 2 School of Public Health and Family Medicine, Falmouth Building, University of Cape Town, Anzio Rd, Observatory, Cape Town, South Africa; 3 Institute for Women’s Health, University College London, Huntley St, London, UK

**Keywords:** Asphyxia, Neonate, Hypoxic ischemic encephalopathy, Electroencephalogram, aEEG, Prognostic

## Abstract

**Background:**

An early clinical score predicting an abnormal amplitude-integrated electroencephalogram (aEEG) or moderate-severe hypoxic ischemic encephalopathy (HIE) may allow rapid triage of infants for therapeutic hypothermia. We aimed to determine if early clinical examination could predict either an abnormal aEEG at age 6 hours or moderate-severe HIE presenting within 72 hours of birth.

**Methods:**

Sixty infants ≥ 36 weeks gestational age were prospectively enrolled following suspected intrapartum hypoxia and signs of encephalopathy. Infants who were moribund, had congenital conditions that could contribute to the encephalopathy or had severe cardio-respiratory instability were excluded. Predictive values of the Thompson HIE score, modified Sarnat encephalopathy grade (MSEG) and specific individual signs at age 3–5 hours were calculated.

**Results:**

All of the 60 infants recruited had at least one abnormal primitive reflex. Visible seizures and hypotonia at 3–5 hours were strongly associated with an abnormal 6-hour aEEG (specificity 88% and 92%, respectively), but both had a low sensitivity (47% and 33%, respectively). Overall, 52% of the infants without hypotonia at 3–5 hours had an abnormal 6-hour aEEG. Twelve of the 29 infants (41%) without decreased level of consciousness at 3–5 hours had an abnormal 6-hour aEEG (sensitivity 67%; specificity 71%). A Thompson score ≥ 7 and moderate-severe MSEG at 3–5 hours, both predicted an abnormal 6-hour aEEG (sensitivity 100 vs. 97% and specificity 67 vs. 71% respectively). Both assessments predicted moderate-severe encephalopathy within 72 hours after birth (sensitivity 90%, vs. 88%, specificity 92% vs. 100%). The 6-hour aEEG predicted moderate-severe encephalopathy within 72 hours (sensitivity 75%, specificity 100%) but with lower sensitivity (p = 0.0156) than the Thompson score (sensitivity 90%, specificity 92%). However, all infants with a normal 3- and 6-hour aEEG with moderate-severe encephalopathy within 72 hours who were not cooled had a normal 24-hour aEEG.

**Conclusions:**

The encephalopathy assessment described by the Thompson score at age 3–5 hours is a sensitive predictor of either an abnormal 6-hour aEEG or moderate-severe encephalopathy presenting within 72 hours after birth. An early Thompson score may be useful to assist with triage and selection of infants for therapeutic hypothermia.

## Background

Intrapartum fetal hypoxia followed by hypoxic ischemic encephalopathy (HIE) is a common cause of potentially avoidable brain injury in term infants [1,2]. The incidence of HIE in developed countries is estimated to be 1.5 per 1,000 live births [3]. Estimates in developing countries range from 2.3–26.5 per 1,000 live births [4,5]. A recent meta-analysis found that therapeutic hypothermia commenced by age 6 hours for infants with moderate or severe (moderate-severe) HIE, significantly reduces death or disability: in three studies an abnormal amplitude integrated electro-encephalogram (aEEG) was required as an additional criterion for cooling [6].

Moderate-severe HIE typically presents with worsening clinical signs after the first 1.5–18 hours and then a slow improvement after 4–5 days [7]. Early identification of infants at risk of developing moderate-severe encephalopathy is crucial: experimental studies emphasize that the sooner hypothermia is started, the better the therapeutic effect [8,9]. During the first 6 hours after birth, a bi-parietal aEEG is the most sensitive and specific single indicator of long-term outcome after HIE [10-12]. In normothermic infants, a normal aEEG during the first 6 hours after birth has a sensitivity of 89% and a positive predictive value (PPV) of 96.2% for a normal long-term outcome [10]. Shalak et al. defined a modification of Sarnat’s [7] encephalopathy grading (the modified Sarnat encephalopathy grade) [13]. They showed that the presence of at least one clinical sign of moderate-severe encephalopathy occurring in at least three of six separate components during the first 12 hours after birth, had a similar sensitivity but lower specificity for prediction of an abnormal outcome at discharge than a severely abnormal fronto-parietal aEEG. Importantly, Shalak found that a combination of both mild and moderate encephalopathic clinical signs identified infants with an abnormal outcome at discharge with a sensitivity of 100%. This is in keeping with our knowledge of the evolving nature of HIE [7,14] and it suggests that infants can subsequently develop moderate-severe encephalopathy following initial signs of mild encephalopathy.

The Thompson score is derived from nine aspects of the neurological examination of infants with HIE: the total score ranges from 0–22 and the kappa coefficient is 0.87 [14]. This score allows a more precise description of infants than “mild”, “moderate” or “severe” and recognizes the prognostic significance of mixed signs within these three categories. In normothermic infants, a maximum score > 10 during the first 7 days of life, predicts an abnormal outcome with 100% sensitivity and 61% specificity [14]. A robust early clinical score that accurately predicts an abnormal aEEG by 6 hours after birth would allow rapid triage of specific babies for therapeutic hypothermia. There are, however, no published data on the prognostic value of a Thompson score obtained < 6 hours after birth.

We studied a group of infants who were evaluated for therapeutic hypothermia with the primary objective of determining the threshold Thompson score at age 3–5 hours that predicted an abnormal 6-hour aEEG.

The secondary objectives were to determine the following:

i.) The predictive value of modified Sarnat encephalopathy grading (MSEG) at age 3–5 hours for an abnormal 6-hour aEEG;

ii.) The predictive value of specific clinical signs at age 3–5 hours for an abnormal 6-hour aEEG. The specific clinical signs we analyzed were those used as entry criteria in the cooling trials with abnormal aEEG as an additional criterion [15-17]. These clinical signs included decreased level of consciousness, visible seizures, hypotonia, and abnormal reflexes;

iii.) The ability of the Thompson score threshold to predict moderate-severe encephalopathy presenting within 72 hours after birth.

## Methods

This prospective cohort study was approved by the University of Cape Town Health Sciences Faculty Human Research Ethics Committee and conforms to the principles of the 2008 Declaration of Helsinki [18]. Informed parental consent was obtained. The study was performed between June 2008 and March 2009 at three hospitals in Cape Town: Groote Schuur Hospital, a tertiary hospital and New Somerset Hospital and Mowbray Maternity Hospital, both secondary hospitals. Recruitment at New Somerset Hospital only commenced in September 2008.

### Study sample

Infants ≥ 36 weeks gestation and birth weight ≥ 2000 g, with signs of encephalopathy after age 10 minutes but before age 5 hours were consecutively recruited using similar criteria for intrapartum hypoxia to those described in the cooling trial by Shankaran et al. [19]. Infants were included if they had one of the following signs suggesting intrapartum hypoxia: i) A base deficit ≥ 16 mmol/l in the first hour of life on cord or infant arterial blood, *or* ii) An abnormal intra-partum course (eg: abnormal fetal heart rate, cord prolapse, uterine rupture, maternal haemorrhage; maternal trauma; maternal seizures; shoulder dystocia; maternal cardiorespiratory arrest, meconium-stained liqor or prolonged second stage) and either a 10-minute Apgar score < 7 or continued respiratory support at 10 minutes. A 10-minute Apgar score < 7 was accepted as it corresponded with the need for continued respiratory support.

Exclusion criteria were any of the following: infection at birth, chorioamnionitis with prolonged rupture of membranes, chromosomal syndromes, cerebral malformations, lethal congenital malformations, neonatal abstinence syndrome, metabolic encephalopathies, moribund infants where demise was imminent or infants with severe cardio-respiratory instability requiring high dose inotropes, fractional inspired oxygen ≥ 0.8, or high frequency oscillatory ventilation (HFOV).

Sample size calculations were based on data from a previous analysis of HIE in our region [14]. We estimated that 33% of eligible infants would have a normal 6-hour aEEG, that 50–70% of these infants would have a Thompson score below threshold and that 2–10% of the infants with an abnormal aEEG would have a Thompson score below threshold. We expected to recruit 60 infants within 12 months. Fisher’s exact analysis of 2×2 tables populated with our estimates and a sample size of 60, yielded p-values ranging from 0–0.0026. According to Buderer’s formula for sample size calculation for diagnostic tests [20], if the prevalence of abnormal aEEG at 6 hours is expected to be 67% and the Thompson score threshold is expected to have a minimum sensitivity of 90%, a minimum sample size of 51 is required if precision is set at 10%. We therefore recruited 60 infants to allow for a margin of error.

### Neurological assessment

A clinical assessment was performed after initial stabilization. A Thompson score was determined [14], and the encephalopathy was graded as mild, moderate or severe according to MSEG [13]: encephalopathy was defined as the presence of one or more abnormal signs in at least three of the following six categories; level of consciousness, spontaneous activity, posture, tone, primitive reflexes (suck or Moro), and autonomic nervous system (pupils, heart rate, or respiration). The grades were defined as follows:

*Mild:* hyperalert, normal tone and activity, exaggerated moro, normal autonomic function;

*Moderate:* lethargic, decreased activity, distil flexion, hypotonia, weak primitive reflexes, constricted pupils, bradycardia or periodic breathing;

*Severe*: stupor/coma, decerebrate posture, absent spontaneous activity, flaccid, absent primitive reflexes, non-reactive pupils or apnoea.

The grade with the most corresponding signs was assigned but if signs were equally distributed, the grade was based on the level of consciousness. Infants with seizures were graded as “moderate” unless severe signs predominated. These assessments were performed at 1 hour (in those presenting early enough), 3–5, 6, and 24 hours after birth and were continued daily until the tenth day or discharge, whichever occurred first. The assessments were performed by the attending pediatric residents or specialists who had received training in performing the Thompson score. After the clinical assessment, an aEEG was obtained on all infants and continuously recorded on a BrainZ BRM2 monitor (BrainZ Instruments Ltd, Auckland, NZ). The recording was continued until the single channel bi-parietal trace remained normal for 24 hours, or until 96 hours, or until the death of the infant, whichever occurred first. In addition to the biparietal aEEG, the BrainZ monitor records the bilateral raw EEG and bilateral aEEG between parietal and central electrodes. Two neonatologists (MC and NJR) with experience in aEEG interpretation and who were blind to the neurological assessments, reviewed the recordings offline. The assessors provided consensus opinion on the category of the bi-parietal background aEEG trace and the presence of seizures at age 3, 6, 24, 48, 72, and 96 hours.

The aEEG recording was classified by background voltage pattern according to the most severely abnormal trace at each of the assessment times ± 30 minutes. The classification was based on the modified system proposed by Hellström-Westas [21]. Five different patterns were described:

1. *Continuous normal voltage (CNV):* Continuous and variable activity with minimum voltage of 5–10 μV and maximum voltage of 10–50 μV.

2. *Discontinuous normal voltage (DNV):* Discontinuous activity with variable minimum amplitude - predominantly below 5 μV, and maximum amplitude above 10 μV.

3. *Burst suppression (BS):* Discontinuous activity with minimum amplitude without variability at < 5 μV and bursts predominantly with amplitude ≥ 25 μV.

4. *Continuous low voltage (CLV):* Continuous and variable activity with maximum amplitude below 10 μV and minimum amplitude around or below 5 μV.

5. *Flat trace (FT):* Primarily inactive (isoelectric) trace with both maximum and minimum background activity below 5 μV.

Seizures on aEEG were defined by an abrupt rise in the minimum and maximum amplitude, confirmed on raw EEG showing repetitive spikes or sharp-wave activity with duration of at least 10 seconds.

The aEEG recordings were further graded as follows:

a) *Normal:* Background activity was CNV and EEG seizures were absent.

b) *Abnormal:* EEG seizures were present or background activity was DNV, BS, CLV or FT. A subgroup with severely abnormal background activity was defined as recordings showing BS, CLV or FT.

### Medical management

All infants received routine monitoring and clinical care. The infants with seizures or an abnormal aEEG were treated with hypothermia within 6 hours of birth. These infants were cooled to a core temperature of 34°C for 72 hours using gel-packs according to the limits of previously described basic cooling methods [22] and they were re-warmed at 0.2°C/hour. All cooled infants were sedated with intravenous (IV) Phenobarbital 20 mg/kg. Morphine 8 μg/kg/hour IV was given if infants were restless or agitated during cooling. Seizures were treated with a second dose of IV Phenobarbital 20 mg/kg. Persistent seizures were treated with Midazolam up to 0.1 mg/kg/hour IV, followed by IV Lignocaine if required.

### Data collection and analysis

In addition to the clinical neurological assessments and aEEG, further data collection included perinatal characteristics, morbidity and short-term outcomes. The data included; maternal age; maternal HIV and Syphilis status; pregnancy complications including hypertension, hemorrhage, thyroid disease and diabetes; intrapartum complications including fetal heart rate abnormalities, cord prolapse, uterine rupture, maternal seizures, shoulder dystocia, maternal hemorrhage, meconium-stained liquor and prolonged second stage; delivery mode; infant characteristics at birth; resuscitation at birth; blood gas within 1 hour of birth; nosocomial sepsis; continuous positive airway pressure (CPAP); mechanical ventilation; inotropic support; pulmonary air leak; length of stay and death.

Data were analyzed with Stata 12 (Stata Corporation, Texas USA). Receiver operating characteristic (ROC) curve analysis was used to determine the threshold Thompson score that predicted an abnormal aEEG. The diagt module was used to calculate the sensitivity, specificity and likelihood ratio (LR) for the threshold Thompson score and moderate-severe encephalopathy at age 3–5 hours to predict an abnormal 6-hour aEEG. Inclusion in one group did not prevent inclusion in the other. The specific clinical signs utilized in the cooling trials were analyzed using the same method. The short-term outcomes and morbidity were compared between infants with or without the threshold Thompson score. The Chi-square or Fisher’s exact tests were used for categorical comparisons. The t-test and the Wilcoxon rank-sum tests were used to compare parametric and non-parametric continuous variables respectively.

To determine the extent to which earlier examinations differed from later assessments in their ability to predict an abnormal 6-hour aEEG, we performed post-hoc analysis of the sub-group of infants with a 1-hour clinical assessment and an aEEG at both 3 and 6 hours. ROC curve analysis was used to determine the threshold Thompson score at 1 and 3–5 hours predictive of an abnormal aEEG at 3 *and/or* 6 hours. Sensitivity, specificity and LR were calculated for moderate-severe encephalopathy and also for the most sensitive Thompson score thresholds at 1 and 3–5 hours.

We also determined the ability of early clinical and aEEG assessment to predict moderate-severe encephalopathy presenting within 72 hours after birth. Predictive values for a Thompson score ≥ 7 at 3–5 hours, moderate-severe encephalopathy at 3–5 hours, abnormal aEEG at 6 hours and abnormal aEEG at 3 *and/or* 6 hours were calculated. The McNemar test was used to confirm significant differences in sensitivity and specificity. All statistical tests are 2-sided at alpha = 0.05.

## Results

There were 80 infants with entry criteria: 20 met exclusion criteria and the remaining 60 infants were recruited. Eight infants (13%) died before discharge. Forty-one infants (68%) were cooled. Cooling was continued for 72 hours except in the six infants who died during cooling. The perinatal characteristics are shown in Table 1. The mother with antenatal syphilis was included because she completed treatment more than a month before delivery and the infant had no signs of congenital infection. The majority of infants (93%) were inborn. Despite the low number of outborn infants, 12 of the 60 infants (20%) did not have a cord or arterial blood gas within the first hour of life. A clinical neurological assessment was performed in all 60 infants at 3–5 hours (3.1 ± 0.4 hours) and only two infants had this assessment performed after 4 hours. Recordings of the 6-hour aEEG were available for 60 infants but a 3-hour aEEG was available for only 50 infants; 43 of these infants also had a 1-hour clinical assessment.

**Table 1:** Perinatal characteristics and short-term neonatal outcomes

**Characteristics and short-term outcomes**	**n (%) / mean (+/-SD) median (IQR**
Maternal Baseline Characteristics, n (%)	60 (100)
Median maternal age, years (IQR)	23 (20 – 26)
HIV negative (Unknown in 3)	42 (70)
Antenatal Syphillis (treated)	1 (2)
Pregnancy complications^a^	11 (18)
Intrapartum complications^b^	54 (90)
Emergency Caesarean section	24 (40)
Normal Vertex Delivery	26 (43)
Forceps / ventouse	10 (17)
Infant baseline characteristics, n (%)	60 (100)
Birth weight, g	3167 (± 517)
Male gender	34 (57)
Outborn	4 (7)
Median Apgar score (IQR)	
1 minute	3 (1 – 4)
5 minute	5 (4 – 6)
10 minute	6 (5 – 7)
Resuscitation	
Chest Compressions	19 (32)
Adrenaline given	6 (10)
Continued respiratory support at 10 minutes	47 (78)
Cord blood gas done	19 (32)
Arterial Blood gas in 1^st^ hour / Cord Gas done	48 (80)
Worst pH in 1^st^ hour (n=48)	7.0 (± 0.2)
Worst Base Deficit in 1^st^ hour	17.8 (± 4.4)

^a^ Includes hypertension, haemorrhage, thyroid disease and diabetes.

^b^ Includes Abnormal fetal heart rate, cord prolapse, uterine rupture, maternal seizures, shoulder dystocia, maternal haemorrhage, meconium-stained liquor and prolonged second stage.

### Clinical signs at 3–5 hours to predict an abnormal 6-hour aEEG

**Figure 1:**
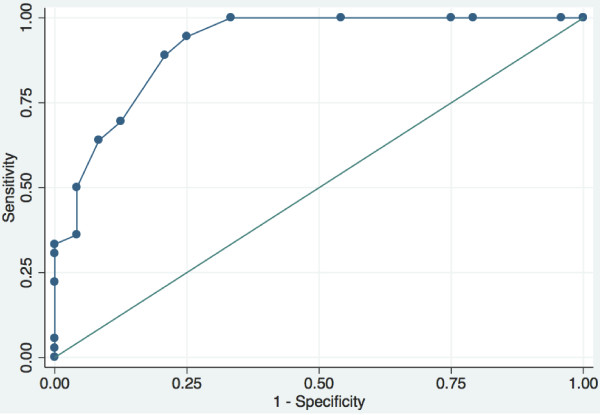
ROC curve: the Thompson score at 3–5 h to predict an abnormal 6-hour aEEG.

The ROC curve for the Thompson score at age 3–5 hours to predict an abnormal 6-hour aEEG (Figure 1) had an area under the curve (AUC) of 0.92 (95% confidence interval (CI) 0.84–0.99). The sensitivity, specificity and LR at different cut points are shown in Table 2. The Thompson score with a sensitivity of 100% and the highest specificity was a score of ≥ 7. The sensitivity, specificity and LR of the Thompson score at a cutoff of ≥ 7 vs. moderate-severe encephalopathy are shown in Table 3. There were no significant differences between the predictive values for these two assessment methods.

**Table 2:** ROC detail - Thompson score at 3 – 5 hours to predict an abnormal 6-hour aEEG

**Thompson score cut point**	**Sensitivity**	**Specificity**	**Correctly classified**	**LR+**	**LR-**
≥ 2	100%	0.00%	60.00%	1.0000	
≥ 3	100%	4.17%	61.67%	1.0435	0.0000
≥ 4	100%	20.83%	68.33%	1.2632	0.0000
≥ 5	100%	25.00%	70.00%	1.3333	0.0000
≥ 6	100%	45.83%	78.33%	1.8462	0.0000
≥ 7	100%	66.67%	86.67%	3.0000	0.0000
≥ 8	94.44%	75.00%	86.67%	3.7778	0.0741
≥ 9	88.89%	79.17%	85.00%	4.2667	0.1404
≥ 10	69.44%	87.50%	76.67%	5.5556	0.3492
≥ 11	63.89%	91.67%	75.00%	7.6667	0.3939
≥ 12	50.00%	95.83%	68.33%	12.0000	0.5217
≥ 13	36.11%	95.83%	60.00%	8.6667	0.6667

*LR*: Likelihood ratio, *ROC*: Receiver operating characteristic.

**Table 3:** Prediction of abnormal 6-hour aEEG with different encephalopathy assessment methods at 3 – 5 hours

**Encephalopathy assessment**	**n**		**Abnormal aEEG n (%)**	**Sensitivity (95%CI)**	**Specificity (95%CI)**	**LR + (95%CI)**	**LR - (95%CI)**	**LR Test (Odds ratio) (95%CI)**
Moderate-severe encephalopathy	Yes	42	35 (83)	97%	71%	3.2 ^a^	0.06 ^a^	55.22 ^a^
	No	18	1 (6)	(86 – 100)	(49 – 87)	(1.75 – 5.84)	(0.01 – 0.28)	(8.73 – 349.15)
Thompson score ≥ 7	Yes	44	36 (82)	100%	67%	2.9 ^a^	0.02 ^a^	141.71 ^a^
	No	16	0 (0)	(90 – 100)	(45 – 84)	(1.7 – 5)	(0 – 0.33)	(7.71 – 2603.42)

*LR*: Likelihood ratio, *aEEG*: amplitude-integrated EEG,

^a^ : When zero count cells are present, LR is estimated using a substitution formula: 0.5 is added to all cell frequencies before calculation.

Univariate analysis of individual clinical signs at age 3–5 hours vs. abnormal 6-hour aEEG is shown in Table 4. At age 3–5 hours, all 60 infants had at least one abnormal primitive reflex. Twelve of the 29 infants (41%) *without* a decreased level of consciousness had an abnormal 6-hour aEEG. Hypotonia was a specific but not sensitive predictor for an abnormal aEEG: half of the infants *without* hypotonia (52%) had an abnormal 6-hour aEEG.

**Table 4:** Univariate analysis: Specific clinical signs at 3 – 5 hours as predictors of abnormal 6-hour aEEG

**Predictor**	**n**		**Abnormal aEEG n (%)**	**Sensitivity (95%CI)**	**Specificity (95%CI)**	**LR + (95%CI)**	**LR - (95%CI)**	**LR Test (Odds ratio) (95%CI)**
Decreased LOC	Yes	31	24 (77)	67%	71%	2.29	0.47	4.86
	No	29	12 (41)	(49 – 81)	(49 – 87)	(1.18 – 4.44)	(0.28 – 0.8)	(1.61 – 14.61)
Seizures visible	Yes	20	17 (85)	47%	88%	3.78	0.6	6.26
	No	40	19 (48)	(30 – 65)	(68 – 97)	(1.24 – 11.5)	(0.43 – 0.85)	(1.67 – 23)
Hypotonia	Yes	14	12 (86)	33%	92%	3.38 ^a^	0.74 ^a^	4.59 ^a^
	No	46	24 (52)	(19 – 51)	(73 – 99)	(0.96 – 11.9)	(0.56 – 0.96)	(1.05 – 20.04)
Stretch reflexes abnormal	Yes	40	31(78)	86%	63%	2.3	0.22	10.33
	No	20	5 (25)	(71 – 95)	(41 – 81)	(1.35 – 3.91)	(0.09 – 0.53)	(3.02 – 35.2)
Primitive reflexes abnormal	Yes	60	36 (100)	100%	0%	1.01 ^a^	0.68 ^a^	1.49 ^a^
	No	0	0 (0)	(90 – 100)	(0 – 14)	(0.94 – 1.08)	(0.01 – 32.95)	(0.03 – 77.62)

^a^: When zero count cells are present, LR is estimated using a substitution formula: 0.5 is added to all cell frequencies before calculation.

*LOC*: Level of consciousness, *LR*:Likelihood ratio, *aEEG*: amplitude-integrated EEG.

### Morbidity and short-term outcomes at the threshold Thompson score

Morbidity and short-term outcomes were compared between infants with a Thompson score of ≥ 7 and those with a Thompson score of < 7 at age 3–5 hours. Forty-four of the 60 infants (73%) had a Thompson score ≥ 7. Nosocomial sepsis, pulmonary air leak and pulmonary hypertension were uncommon events and there was no significant difference in the incidence between the groups. More infants in the group with a Thompson score ≥ 7 required CPAP (p=0.036), mechanical ventilation (p=0.002) and inotropic support (p=0.048), than did those with a lower Thompson score. Forty of the 44 infants with a Thompson score ≥ 7 (91%) met criteria for therapeutic hypothermia and were cooled. Only one infant with a Thompson score < 7 was cooled. This infant had an abnormal 3-hour aEEG showing a DNV background, but the background normalized by 6 hours.

### Predicting an abnormal aEEG at 3 and/or 6 hours

Sub-group analysis was performed on the 43 infants who had a 1-hour clinical assessment as well as an aEEG at both 3 and 6 hours. Twenty-eight infants in this sub-group had an abnormal aEEG at 3 *and/or* 6 hours and all of these 28 infants were cooled. The remaining 15 infants were not cooled. ROC curve analysis of the Thompson score at age 1 hour vs. an abnormal aEEG at 3 *and/or* 6 hours determined that the highest predictive Thompson score with a sensitivity of 100% was a score of ≥ 6 (AUC 0.93, 95%CI: 0.86–1.00). This threshold therefore also identified all the infants in this subset who were cooled. However, specificity at this threshold was only 33% and most infants were correctly classified at a Thompson score ≥ 7. ROC curve analysis of the Thompson score at age 3–5 hours vs. an abnormal aEEG at 3 *and/or* 6 hours determined that the highest predictive Thompson score with a sensitivity of 100% was a score of ≥ 5 (AUC 0.91, 95%CI: 0.82–1.00) but specificity at this threshold was only 27%. The sensitivities, specificities and LRs for Thompson score and moderate-severe encephalopathy at 1 and 3–5 hours to predict an abnormal aEEG at 3 *and/or* 6 hours are shown in Table 5.

**Table 5:** Predicting abnormal aEEG at 3 or 6 hours: assessments at 1 and 3 – 5 hours

**Encephalopathy assessment method**	**n**		**Abnormal aEEG n (%)**	**Sensitivity (95%CI)**	**Specificity (95%CI)**	**LR +(95%CI)**	**LR - (95%CI)**	**LR Test (Odds ratio) (95%CI)**
Moderate-severe encephalopathy age 1 hour	Yes	31	25 (81)	89%	60%	2.23	0.18	12.5
	No	12	3 (25)	(72 – 98)	(32 – 84)	(1.19 – 4.2)	(0.06 – 0.56)	(2.71 – 56.89)
Moderate-severe encephalopathy age 3 – 5 hours	Yes	29	26 (90)	93%	80%	4.64	0.09	52
	No	14	2 (14)	(77 – 99)	(52 – 96)	(1.68 – 12.84)	(0.02 – 0.35)	(8.24 – 321.56)
Thompson score ≥ 8 age 3 – 5 hours	Yes	28	25 (89)	89%	80%	4.46	0.13	33.33
	No	15	3 (20)	(72 – 98)	(52 – 96)	(1.61 – 12.38)	(0.04 – 0.4)	(6.16 – 180.87)
Thompson score ≥ 7 age 3 – 5 hours	Yes	31	26 (84)	93%	67%	2.79	0.11	26
	No	12	2 (17)	(77 – 99)	(38 – 88)	(1.35 – 5.74)	(0.03 – 0.43)	(4.67 – 139.13)
Thompson score ≥ 6 age 3 – 5 hours	Yes	34	26 (76)	93%	47%	1.74	0.15	11.38
	No	9	2 (22)	(77 – 99)	(21 – 73)	(1.07 – 2.83)	(0.04 – 0.65)	(2.15 – 57.78)
Thompson score ≥ 5 age 3 – 5 hours	Yes	39	28 (72)	100%	27%	1.37 ^a^	0.06 ^a^	22.3 ^a^
	No	4	0 (0)	(88 – 100)	(8 – 55)	(1 – 1.86)	(0 – 1.07)	(1.11 – 448.4)
Thompson score ≥ 8 age 3 – 5 hours	Yes	30	26 (87)	93%	73%	3.48	0.1	35.75
	No	13	2 (15)	(77 – 99)	(45 – 92)	(1.5 – 8.11)	(0.02 – 0.38)	(6.13 – 201.31)
Thompson score ≥ 7 age 3 – 5 hours	Yes	32	27 (84)	96%	67%	2.76 ^a^	0.08 ^a^	35 ^a^
	No	11	1 (9)	(82 – 100)	(38 – 88)	(1.39 – 5.46)	(0.02 – 0.39)	(5.03 – 243.64))
Thompson score ≥ 6 age 1 hour	Yes	38	28 (74)	100%	33%	1.5 ^a^	0.05 ^a^	29.86 ^a^
	No	5	0 (0)	(88 – 100)	(12 – 62)	(1.05 – 2.14)	(0 – 0.85)	(1.52 – 587.98)
Thompson score ≥ 5 age 1 hour	Yes	39	28 (72)	100%	27%	1.37 ^a^	0.06 ^a^	22.3 ^a^
	No	4	0 (0)	(88 – 100)	(8 – 55)	(1 – 1.86)	( 0 – 1.07)	(1.11 – 448.4)

aEEG: amplitude integrated electro-encephalogram, LR: Likelihood ratio.

^a^ : When zero count cells are present, LR is estimated using a substitution formula: 0.5 is added to all cell frequencies before calculation.

### Neurological short-term outcomes

Comparison of neurological short-term outcomes in infants at or below the threshold Thompson score at 3–5 hours are shown in Table 6. Most infants (86%) with a Thompson score ≥ 7 had an abnormal aEEG at 3 and/or 6 hours. All of the infants with a Thompson score < 7 had a normal 6-hour aEEG, but two of them had an abnormal 3-hour aEEG. The 3-hour aEEG in both infants was DNV and it corrected to a normal background by 6 hours. One of these infants was cooled on the basis of the abnormal aEEG, but in the other infant the aEEG had normalized by the time cooling was considered and cooling was not commenced. None of the infants with a Thompson score < 7 had seizures. Ninety-eight percent of infants (43/44) with a Thompson score ≥ 7 developed moderate-severe encephalopathy within 72 hours of birth, but the majority of these infants (41/43) were cooled.

**Table 6:** Short-term neurological outcomes

**Outcome**	**Whole cohort n = 60 (100%)**	**Thompson < 7 n = 16 (100%)**	**Thompson ≥ 7 n = 44 (100%)**
Mild encephalopathy ^a^	12 (20)	11 (69)	1 (2) *
Moderate encephalopathy ^a^	27 (45)	5 (31)	22 (50) *
Severe encephalopathy ^a^	21 (35)	0	21 (48) *
aEEG or Clinical Seizure	35 (58)	0	35 (80) *
Mean core temperature age 3 hours (±SD)	35.3 (±1.1)	36.3 (±0.4)	34.9 (±1.0) *
aEEG normal at 6 and 24 hours	23 (38)	16 (100)	7 (16) *
aEEG abnormal age 3 hours (n=50)	33 (66)	2(13)	31(71) *
aEEG abnormal age 6 hours	36 (60)	0	36 (82) *
aEEG abnormal age 3 or 6 hours	40 (67)	2(13)	38 (86) *
aEEG severely abnormal age 6 hours	25 (42)	0	25 (57) *
aEEG severely abnormal age 24 hours	18 (30)	0	18 (41) **
Dead or aEEG severely abnormal age 48 hours	12 (20)	0	12 (27) *†*
Maximum Thompson score ≥ 15	21 (35)	0	21 (48) **
Dead or maximum Thompson Score ≥ 15	22 (37)	0	22 (50) *
Dead	8 (13)	0	8 (19) *‡*
Median age at death, d (Range) (n=8)	2 (1.5 – 5)	-	2 (1.5 – 5)
Thompson score normal (0) by day 7	22 (42)	13 (81)	9 (25) *

LOC: Level of consciousness, aEEG: amplitude-integrated EEG.

^a^: Highest modified Sarnat encephalopathy grade recorded in the first 72 hours.

* p<0.0001, ** p=0.001, † p=0.025, ‡ p=0.09.

The predictive values of MSEG, Thompson score and aEEG for moderate-severe encephalopathy within 72 hours are shown in Table 7. The differences between the predictive values for the Thompson score and MSEG were not significant, but the sensitivity of the Thompson score (90%) was significantly higher (Exact McNemar p=0.0156) than that of the 6-hour aEEG (75%).

**Table 7:** Prediction of moderate-severe HIE presenting in the first 72 hours after birth

**Encephalopathy assessment**	**n**		**moderate-severe HIE n (%)**	**Sensitivity (95%CI)**	**Specificity (95%CI)**	**LR + (95%CI)**	**LR - (95%CI)**	**LR Test (Odds ratio) (95%CI)**
aEEG abnormal age 6 hours	Yes	36	36 (100)	75%	100%	19.4 ^a^	0.27 ^a^	73 ^a^
	No	24	12 (50)	(60 – 86)	(74 – 100)	(1.3 – 294.9)	(0.16 – 0.43)	(4 – 1325)
Moderate-severe encephalopathy age 3 – 5 hours	Yes	42	42 (100)	88%	100%	23 ^a^	0.1 ^a^	163.5 ^a^
	No	18	6 (33)	(75 – 95)	(74 – 100)	(1.5 – 342.4)	(0.1 – 0.3)	(8.6 – 3106.8)
Thompson score ≥ 7 age 3 – 5 hours	Yes	44	43 (98)	90%	92%	7.7 ^a^	0.1 ^a^	60.6 ^a^
	No	16	5 (31)	(77 – 97)	(62 – 100)	(1.7 – 34.8)	(0.1 – 0.3)	(8.9 – 413.1)
aEEG abnormal age 3 hours and/or 6 hours	Yes	40	39 (98)	81%	50%	1.6 ^a^	0.4 ^a^	4.2 ^a^
	No	10	9 (90)	(67 – 91)	(1 – 99)	(0.5 – 5)	(0.1 – 1.4)	(0.4 – 44.5)

*aEEG*: amplitude integrated electro-encephalogram, *LR*: Likelihood ratio, *CI*: Confidence Interval, *LOC*: Level of consciousness.

*HIE*: Hypoxic ischaemic encephalopathy.

^a^: When zero count cells are present, LR is estimated using a substitution formula: 0.5 is added to all cell frequencies before calculation.

Moderate-severe encephalopathy during the first 72 hours occurred in five of 16 infants (31%) with a Thompson score < 7 at 3–5 hours. All five infants had a normal aEEG at both 6 and 24 hours and none of these infants were cooled. Thirty-three percent of infants (6/18) *without* moderate-severe encephalopathy at 3–5 hours, developed moderate-severe encephalopathy within 72 hours. However, 5 of the 6 infants had a normal aEEG at 24 hours: the sixth infant had an abnormal aEEG at 6 hours and was cooled. In comparison, 50% of the infants with a normal aEEG at 6 hours (12/24) developed moderate-severe encephalopathy within 72 hours, but three of these infants had an abnormal aEEG at 3 hours. A 3-hour aEEG was not available in one infant and one infant was cooled on the basis of suspected clinical seizures. The remaining seven infants with moderate-severe encephalopathy by 72 hours, but normal aEEG at both 3 and 6 hours, were not cooled and all had a normal aEEG at 24 hours.

Overall, within the entire cohort of 60 infants there were eight infants with moderate-severe encephalopathy by 72 hours who were not cooled: all of these infants had a normal aEEG at both 6 and 24 hours and the median day of discharge (with normal nutritive suck) was day 5 (IQR day 4–7). Neurological examination on day 7 or at discharge, which ever occurred first, was normal in five infants. The Thompson score in the other three infants ranged from 1–2.

## Discussion

Our data suggest that a Thompson score of ≥ 7 at age 3–5 hours identifies all infants with an abnormal 6-hour aEEG with a specificity of 67%. The presence of moderate-severe encephalopathy at the same age had similar specificity and sensitivity but failed to identify one infant with an abnormal 6-hour aEEG. Individual clinical signs previously used as entry criteria in clinical cooling trials had low sensitivity and/or low specificity for an abnormal 6-hour aEEG. A Thompson score ≥ 5 at 1 *or* 3–5 hours identified all infants with an abnormal aEEG at 3 *and/or* 6 hours but with low specificity. A Thompson score of ≥ 7 at 3–5 hours predicted moderate-severe encephalopathy presenting within 72 hours after birth. However, the hypothermia and the sedating medication received by 83% (40/48) of the infants with moderate-severe encephalopathy may have exaggerated the abnormal signs.

We defined an abnormal aEEG as one that qualifies an infant for cooling according to published protocols [15-17]. This definition allows inclusion of infants with DNV. In a retrospective study of aEEG in cooled vs. normothermic infants, Thoresen et al. defined a normal aEEG as one with CNV *or* DNV [23]. However, following their observation that three of the nine normothermic infants with DNV had a severely abnormal outcome vs. none of the eight cooled infants with DNV, they conceded it was reasonable to cool infants with DNV at 3–6 hours.

The predictive values for an abnormal outcome are lower for an aEEG at 3 vs. 6 hours [12] and an early abnormal assessment might inappropriately select infants for cooling therapy who were destined to be normal without cooling. Post-hoc analysis of the subgroup of infants with 1-hour assessments (Table 6) showed that an earlier Thompson score was more sensitive but less specific in predicting an abnormal aEEG at 3 *and/or* 6 hours (and therefore the need for cooling). If a single Thompson score threshold to be used at 1 *or* 3–5 hours is to be defined, a score of ≥ 7 had the best overall combination of high sensitivity and high diagnostic odds ratios of 35 and 26 respectively. The Thompson score of ≥ 5 at 1 *or* 3–5 hours identified all infants with an abnormal aEEG at 3 *and/or* 6 hours (and all the infants who were cooled). Although this threshold may be suitable to identify infants for referral or further assessment, the low specificity and LR makes it unsuitable as a sole indication for cooling.

Several cooling trials required the presence of a decreased level of consciousness and/or hypotonia as minimum evidence of moderate or severe encephalopathy before further assessment [15-17,24,25]. In our study, a significant proportion of the infants without either a decreased level of consciousness or hypotonia at 3–5 hours had an abnormal 6-hour aEEG. Our data suggest that the absence of specific individual clinical signs should not be grounds for excluding infants from aEEG assessment or cooling therapy.

Shalak et al. studied 50 infants with suspected intra-partum hypoxia [13]. They determined the ability of the moderate-severe encephalopathy at age 5±3 hours and an abnormal fronto-parietal aEEG acquired within an hour of the examination, to predict moderate-severe encephalopathy persisting until the fifth day. The clinical assessments were performed slightly later than in our study and the aEEG position was fronto-parietal, but similar to our study, they found that 23% of infants *without* all the criteria for moderate-severe encephalopathy had an abnormal aEEG and progressed to moderate encephalopathy persisting on the fifth day. The sensitivity and specificity of early moderate-severe encephalopathy to predict moderate-severe encephalopathy persisting to the 5^th^ day were both 78%, but the combination of an abnormal aEEG and encephalopathy including clinical signs of both mild and moderate encephalopathy increased the specificity to 94%. In our study, only one of the infants (6%) without all the criteria for moderate-severe encephalopathy at 3–5 hours had an abnormal aEEG at 6 hours and developed persistent moderate-severe encephalopathy by the fifth day. By comparison, none of the infants with a Thompson score < 7 had an abnormal aEEG at 6 hours, and moderate-severe encephalopathy did not persist to the fifth day in any of those infants. One infant with a Thompson score < 7 was cooled, but data from a secondary analysis of the CoolCap cooling trial data suggest that the hypothermia is unlikely to have influenced the improvement in grade of encephalopathy in this infant [26].

Sarkar et al. have questioned whether a 6-hour aEEG should be used to identify infants for cooling [27]. In a retrospective analysis, they reported that 13 of 24 infants with a normal aEEG had abnormal magnetic resonance imaging (MRI), but the rates of abnormal outcome were similar between the cooled and normothermic groups. In a prospective study, Shankaran et al. found that a fronto-parietal aEEG at < 9 hours did not enhance the predictive value of HIE grade at < 6 hours [28]. Twelve of 71 infants with moderate HIE had a CNV background at < 9 hours and three of these infants had an abnormal outcome. The data of both Sarkar and Shankaran suggests that a normal aEEG in the first 6–9 hours does not guarantee a normal outcome. However it is still unclear whether cooling infants with a normal aEEG is beneficial.

The Thompson score ≥ 7 at 3–5 hours identified more infants with moderate-severe encephalopathy than did the 6-hour aEEG, but the additional infants identified by the Thompson score were predominantly those who were not cooled. The presence of CNV or DNV background voltage on aEEG at 24 hours is significantly associated with normal outcomes in both cooled and normothermic infants[23,29]. The finding of a normal 24-hour aEEG, very low or normal Thompson scores by day 7, and a median discharge age of 5 days, in the infants with moderate-severe encephalopathy who were not cooled suggests that these infants did not require cooling. Thus, although clinical assessment with either MSEG or the Thompson score identified significantly more infants with moderate-severe encephalopathy, this may not be clinically significant.

This study has several limitations. The exclusion of sick infants may alter the sensitivity and specificity of the Thompson score – the findings of this study cannot be applied to the infants whom were excluded. It is a significant weakness of the study that we did not determine the kappa coefficient for the multiple assessors (raters) in this particular study. However, all clinicians were trained, the kappa coefficient for two raters of the Thompson score in a previous study at one of the study sites (Groote Schuur Hospital) is known to be high [14], and the use of multiple raters replicates the setting in which this particular diagnostic approach would ultimately be implemented. The lack of data from age 1 hour in all infants compromised the strength of our conclusions regarding very early assessments. Although the primary objective was to study the prediction of an abnormal 6-hour aEEG, the availability of MRI or long-term follow up data would have allowed further validation and interpretation of the threshold Thompson scores, particularly in the infants with moderate-severe encephalopathy who were not cooled. A further important limitation is that the use of phenobarbitone and morphine may have resulted in overdiagnosis of moderate-severe encephalopathy in the infants who were cooled.

The strengths of our study are that we prospectively recruited infants with all grades of HIE and we obtained at least one clinical and one aEEG assessment by age 6 hours. We compared previously published neurological assessments to a validated aEEG assessment method as a gold standard. We blinded the clinicians’ assessments by comparing their clinical assessment with a later aEEG recording and neonatologists who were blind to the clinical assessments subsequently read the aEEG recordings.

## Conclusions

We have shown that, in a cohort of term infants with all grades of HIE, a Thompson score of ≥ 7 or the presence of a moderate-severe modified Sarnat encephalopathy grade at age 3–5 hours both independently predict an abnormal aEEG at 6 hours, with similar predictive values. A Thompson score of ≥ 7 at age 3–5 hours was a more sensitive predictor of moderate-severe HIE presenting within 72 hours than a 6-hour aEEG. This data may assist in providing a threshold for intervention and/or benchmarking early neurological assessments of infants being considered for neuroprotective hypothermia. A Thompson score ≥ 5 at 1 or 3 hours identified all infants with an abnormal aEEG at 3 *and/or* 6 hours – this may be an appropriate threshold to guide early referral to cooling centers for further assessment. An abnormal 6-hour aEEG can occur in infants without decreased level of consciousness or hypotonia and these single signs may not be appropriate as minimum criteria for cooling. The Thompson score thresholds we have identified should be validated in larger studies with long-term outcomes and MRI. Further research should investigate long-term outcomes of infants with signs of moderate HIE but normal aEEG who are not cooled.

## Abbreviations

(aEEG): Amplitude-integrated electroencephalogram; (AUC): Area under the curve; (BS): Burst suppression; (CI): Confidence interval; (CLV): Continuous low voltage; (CNV): Continuous normal voltage; (CPAP): Continuous positive airway pressure; (DNV): Discontinuous normal voltage; (FT): Flat trace; (HIE): Hypoxic ischemic encephalopathy; (LR): Likelihood ratio; (MRI): Magnetic resonance imaging; (MSEG): Modified Sarnat encephalopathy grade; (PPV): Positive predictive value; (ROC): Receiver operating characteristic

## Competing interests

The authors declare that they have no competing interests.

## Authors’ contributions

ARH conceived of the study, designed the research protocol, supervised and participated in the research, drafted the manuscript and performed the statistical/graphic analyses. GHS, and LM participated in the design of the study and critically reviewed the draft manuscript. LLL, MSR, YJ, MCH, MC and NRR assisted with acquisition of data and critically reviewed the draft manuscript. NJR participated in the design of the study, assisted with acquisition of data and critically reviewed the draft manuscript. All authors read and approved the final manuscript.

## Authors’ information

Dr Alan R Horn: MBChB, FCPaed, Cert. Neon

Prof George H Swingler: MBChB, FCPaed, PhD

Assoc. Prof Landon Myer: MBChB, PhD

Dr Lucy L Linley MBChB, FCPaed

Dr Moegammad S Raban MBChB, FCPaed, Cert. Neon.

Dr Yaseen Joolay MBChB, FCPaed, Cert. Neon.

Dr Michael C Harrison FRCPCH

Dr Manigandan Chandrasekaran MBBS, MRCPCH

Dr Natasha R Rhoda MBChB, FCPaed, Cert. Neon

Prof Nicola J Robertson FRCPCH, PhD

## Pre-publication history

The pre-publication history for this paper can be accessed here:

http://www.biomedcentral.com/1471-2431/13/52/prepub
